# Adenosine deaminase in the cerebrospinal fluid: a measure of inflammation, not a signature of a pathogen

**DOI:** 10.1055/s-0046-1829152

**Published:** 2026-10-23

**Authors:** Abelardo Q. C. Araújo

**Affiliations:** 1 Universidade Federal do Rio de Janeiro, Instituto de Neurologia Deolindo Couto, Rio de Janeiro RJBrazil; 2 Fundação Oswaldo Cruz, Instituto Nacional de Infectologia Evandro Chagas, Rio de Janeiro RJBrazil; 3 Universidade Federal Fluminense, Programa de Pós-Graduação em Neurologia e Neurociências, Niterói RJBrazil

Some laboratory tests never quite earn their place, and never quite lose it. Adenosine deaminase (ADA) in the cerebrospinal fluid (CSF) has occupied that uncertain territory for decades: inexpensive, rapid, available in almost any hospital laboratory, and ordered on the assumption that a raised value points toward tuberculous meningitis (TBM). The study by Farias et al. 1 in this issue is valuable precisely because it asks what that assumption is worth where the question matters most.

Its principal strength is the rigor of the case definition. Each of the 166 patients with central nervous system (CNS) infection carried a microbiological diagnosis — by multiplex PCR, culture, GeneXpert or Xpert Ultra, cryptococcal antigen, or CSF VDRL — compared with 40 patients whose CSF was normal. The etiologic spectrum is unusually wide: alongside *Mycobacterium tuberculosis* , *Streptococcus pneumoniae* and *Cryptococcus* spp., the cohort encompasses neurosyphilis, arboviral infection, CNS histoplasmosis, and patients carrying two or three simultaneous pathogens. Nearly two thirds of the infected patients, and 72.5% of the controls, were living with HIV. This is the differential faced daily in Brazil, sub-Saharan Africa and South Asia, and almost never the one against which ADA has been assessed.

The performance is modest. At a threshold of 5.5 IU/L, the authors report 61.2% sensitivity and 72.4% specificity for TBM, and, across the infected cohort as a whole, 47.6% sensitivity and 51.2% overall accuracy. One third of the patients with confirmed TBM fell below the cutoff. These values lie beneath the pooled estimates of the largest meta-analysis (sensitivity 0.86, specificity 0.89, 43 studies) 2 and of an earlier synthesis (89% and 91%). 3

On one point the authors are more right than they claim. They present their 5.5 IU/L threshold as unexpectedly low; in fact it reproduces, to the decimal, the optimal cutoff derived by Pannu et al. in 251 patients with TBM against 131 with other CNS infections, where specificity was 60.3% and the 10 U/L threshold yielded 50% sensitivity. Discrimination in that series was better against viral meningoencephalitis than against bacterial or cryptococcal meningitis. 4 Independent replication of a threshold across two continents is a more durable contribution than the novelty claimed.

That the numbers are modest reflects the test more than the study. Two constraints operate. The first is the comparator: pooled estimates derive largely from series contrasting TBM with viral or aseptic meningitis, and the closer the controls approach the true clinical dilemma, the less ADA discriminates. Ekermans et al. found this in the largest series assembled, 3,548 CSF-ADA requests in South Africa, where the area under the curve reached 0.86 yet no threshold proved usable in routine practice, 13 of 92 microbiologically confirmed cases of TBM were missed, and ADA failed to separate confirmed TBM from confirmed pyogenic meningitis. 5 The second is analytical: ADA measurement remains unstandardized, and Giusti and non-Giusti methods, differing platforms, matrix effects and the absence of CSF-specific control material render thresholds essentially local. 5 Cutoffs travel poorly — a limitation of the field rather than of any single laboratory.

The most intriguing observation concerns *Neisseria meningitidis* . Eleven such patients had significantly higher CSF-ADA than controls, and the highest value in the series, reported as 58.5 IU/L, occurred in this subgroup. The authors interpret it with appropriate restraint, noting both the marginal significance and the influence of a single outlier. Their caution is warranted, and so is the interest: the finding accords with the South African observation that pyogenic meningitis and TBM overlap in ADA. 5 Acute purulent meningitis can produce values in the tuberculous range. The elevations noted in CNS histoplasmosis rest on two patients and are best regarded, as the authors regard them, as hypothesis-generating.

Read alongside recent work, the central message comes into focus. In Korea, among all patients with a CSF-ADA ≥ 10 IU/L over a decade, the most frequent diagnosis was hematologic malignancy (26.2%), ahead of TBM (19.0%) and viral meningitis (18.2%), with no significant difference between the malignant and tuberculous groups. 6 In Japan, 276 patients investigated for suspected TBM showed elevated ADA in fungal meningitis, sarcoidosis, lymphoma and autoimmune GFAP astrocytopathy. 7 And in these Arquivos three decades ago, Machado, Livramento and Spina-França measured ADA in 263 CSF samples from patients with AIDS and found comparable elevations across toxoplasmosis, syphilis, cytomegalovirus infection, cryptococcal disease, tuberculosis and lymphoma — diagnoses established largely by serology and antigen detection — with no difference among them, while patients with otherwise unremarkable CSF had normal values. 8 ADA, they concluded, tracks the activity of the associated disease. Farias et al. have now confirmed that judgment with modern microbiology, which is no small thing.

The biology is coherent: ADA reports intrathecal T cell activation and purine turnover — the intensity of the immune response rather than its cause. The clinical practice guideline of the Tuberculous Meningitis International Research Consortium reaches the corresponding position: certainty of evidence is very low and the recommendation weak; elevated concentrations are not specific for TBM, though they may usefully prompt more definitive testing; and ADA should not replace Xpert MTB/RIF Ultra or mycobacterial culture. 9 Nor is Ultra a rule-out test, with 76.5% sensitivity against the uniform case definition among HIV-positive Ugandan adults and a negative predictive value of 92.7%. 10 In a patient with HIV, cryptococcal antigen testing comes first.

Two refinements would help future studies. ADA should be evaluated against the uniform case definition for TBM 11 rather than against non-inflammatory controls, and reported as likelihood ratios and incremental value over routine CSF variables rather than as a single institutional threshold. Beyond that, ADA merits study for what it plainly is: a quantitative index of intrathecal inflammation, potentially useful for triage and monitoring.

Farias et al. have not rehabilitated CSF-ADA, and a single retrospective series was never going to. What they have done is describe, with a completeness few centers could match, how it behaves across the crowded, HIV-shaped differential of a Brazilian tropical hospital — including etiologies for which no comparable data existed. That is the kind of careful regional observation on which better global guidance is eventually built.
